# Supplementary material for: COVID-19 in Children: Molecular Profile and Pathological Features
Source: Int J Mol Sci. 2023 Nov 25;24(23):16750. doi: 10.3390/ijms242316750 (PMC10706827; doi:10.3390/ijms242316750)
Supplement: Supplementary file 1 [file ijms-24-16750-s001.zip › ijms-2680495-supplementary.pdf]

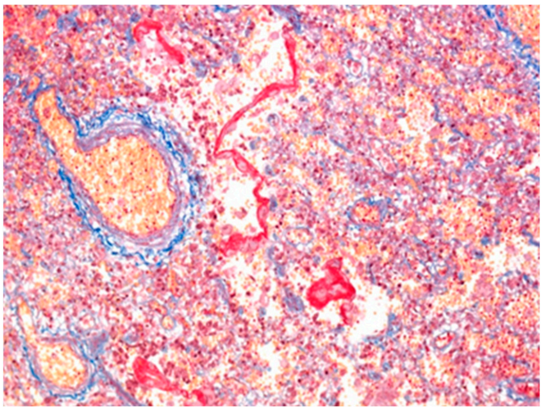

Fig. S1

Figure S1. Changes in the lungs in Patient 1. Tape-like and slightly stained eosinophilic films covering the inside part of the alveoli walls. Staining via trichrome according to Masson's method under x200 magnification.

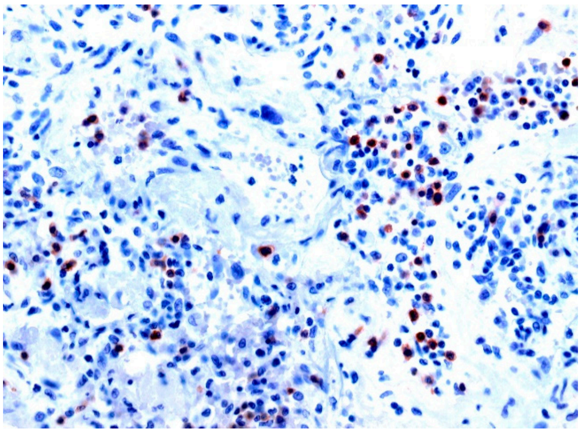

Fig. S2A

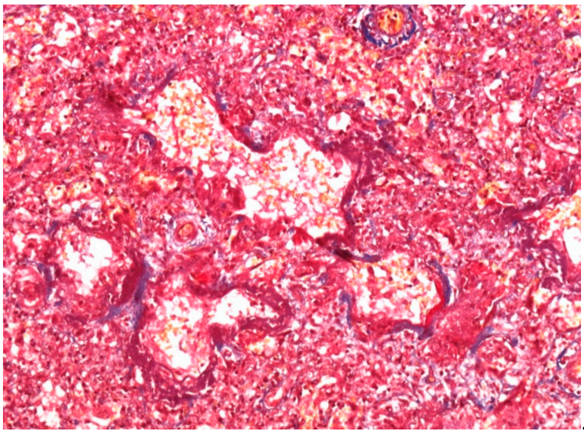

Fig. S2B

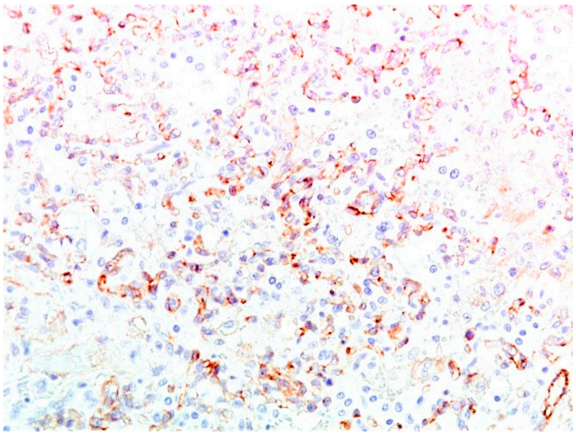

Fig. S2C

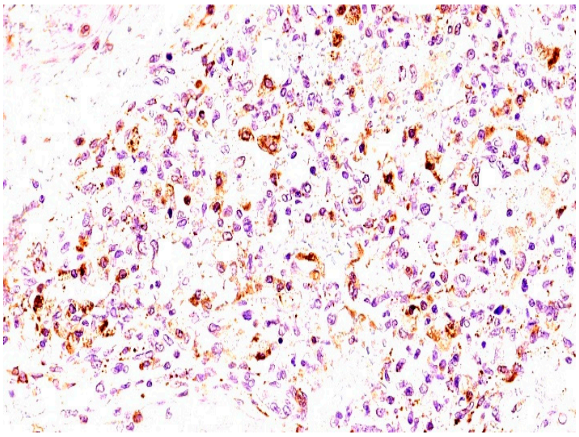

Fig. S2D

Figure S2. Changes in the lungs in Patient 2. (S2A) Inflammatory cell infiltration. IHC staining with antibodies of CD8+ under x400 magnification. (S2B) Dense protein aggregates in the alveoli of all lung tissue and tape-like fibrin masses. Staining via trichrome according to Masson's method under x200 magnification. (S2C) The irregular capillary network, the majority of endothelium cells in the vessels in microcirculation lacked accurate identification. CD31 immunostaining under x400 magnification. (S2D) The acute positive expression of the apoptosis marker in alveolar macrophages and some alveolocytes were noted. CD95 staining under x400 magnification.

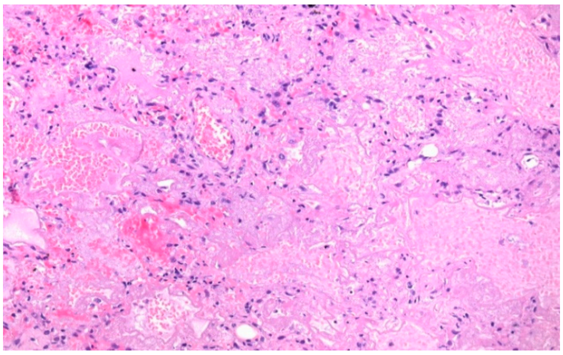

Fig. S3.

Figure S3. Changes in the lungs in Patient 3. Fibrinogenous masses and intra-alveolar hemorrhage, which completely filled the whole volume of the alveoli. H&E under x200 magnification.

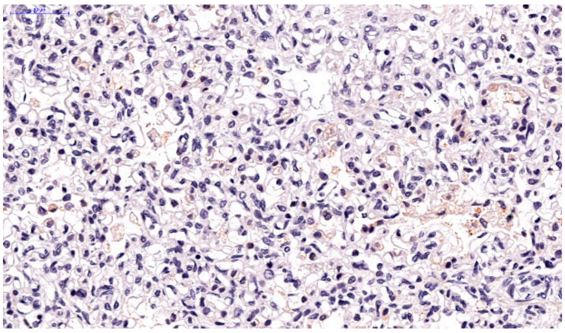

Fig. S4A.

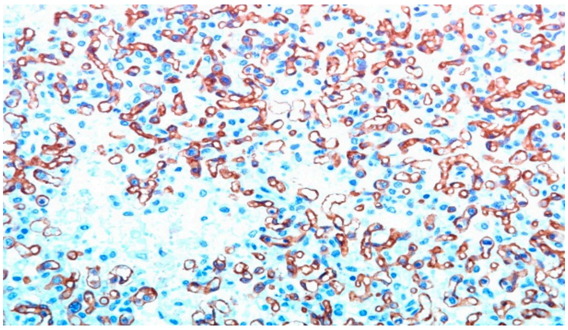

Fig. S4B.

Figure S4. Changes in the lungs in Control Patient. (S4A) The absence of CD 95 expression under x200 magnification. (S4B) The capillary network structures and CD31 staining under x200 magnification.
